# The Pathogenetic Mechanism for Moyamoya Vasculopathy Including a Possible Trigger Effect of Increased Flow Velocity

**DOI:** 10.31662/jmaj.2022-0104

**Published:** 2022-12-19

**Authors:** Takeo Abumiya, Miki Fujimura

**Affiliations:** 1 Department of Neurosurgery, Hokkaido University Graduate School of Medicine, Sapporo, Japan; 2 Department of Neurosurgery, Miyanomori Memorial Hospital, Sapporo, Japan

**Keywords:** Moyamoya disease, Moyamoya syndrome, Moyamoya vasculopathy, Pathogenetic mechanism, Increased flow velocity

## Abstract

Moyamoya disease (MMD), which commonly exhibits moyamoya vasculopathy characterized by chronic progressive steno-occlusive lesions in the circle of Willis with “moyamoya” collateral vessels, has been well known for its unique demographic and clinical features. Although the discovery of the susceptibility gene *RNF213* for MMD revealed the factor for its predominance in East Asians, the mechanisms underlying other predominant conditions (females, children, young to middle-aged adults, and anterior circulation) and lesion formation are yet to be determined. As MMD and moyamoya syndrome (MMS), which secondarily produces moyamoya vasculopathy due to pre-existing diseases, have the same vascular lesions despite differences in their original pathogenesis, they may share a common trigger for the development of vascular lesions. Thus, we herein consider a common trigger from a novel perspective on blood flow dynamics. Increased flow velocity in the middle cerebral arteries is an established predictor of stroke in sickle cell disease, which is often complicated by MMS. Flow velocity is also increased in other diseases complicated by MMS (Down syndrome, Graves’ disease, irradiation, and meningitis). In addition, increased flow velocity occurs under the predominant conditions of MMD (females, children, young to middle-aged adults, and anterior circulation), suggesting a relationship between flow velocity and susceptibility to moyamoya vasculopathy. Increased flow velocity has also been detected in the non-stenotic intracranial arteries of MMD patients. In a pathogenetic overview of chronic progressive steno-occlusive lesions, a novel perspective including the trigger effect of increased flow velocity may provide insights into the mechanisms underlying their predominant conditions and lesion formation.

## Introduction

Moyamoya disease (MMD), which commonly exhibits moyamoya vasculopathy characterized by chronic progressive steno-occlusive lesions in the circle of Willis with “moyamoya” (a hazy puff of smoke in Japanese) collateral vessels, is well known for its unique demographic and clinical characteristics ^(1), (2), (3), (4)^. MMD predominantly occurs in females, children and young to middle-aged adults, and Asians ^(5)^. Steno-occlusive lesions are more common; they are noted to occur earlier in the anterior circulation than in the posterior circulation ^(5)^. MMD is defined as an idiopathic disease without other diseases to induce vascular lesions ^(6), (7)^. Moyamoya vasculopathy associated with pre-existing diseases, such as sickle cell disease (SCD), Down syndrome, neurofibromatosis, Graves’ disease, irradiation, and meningitis, is defined as moyamoya syndrome (MMS) ^(2), (8)^. Ischemic stroke can occur in children and adults with MMD, whereas hemorrhagic stroke predominantly occurs in adults with MMD ^(5)^. In terms of treatment, direct and indirect bypass surgeries have been established to reduce the risk of stroke events ^(9), (10)^. However, no medical treatment has proven effective for stroke prevention in MMD patients. One major reason for the lack of an effective medical treatment may be that the pathogenetic mechanisms of moyamoya vasculopathy have not yet been elucidated in detail. Therefore, further studies to clarify the underlying pathogenetic mechanisms are needed not only for scientific interest, but also for therapeutic development.

As a breakthrough in research on the pathogenetic mechanisms of MMD, *RNF213* in the 17q25-ter region was identified as a susceptibility gene for the onset of MMD ^(11), (12)^. While the frequency of the *RNF213* p.R4810K variant was shown to be approximately 79%-90% in MMD patients in South Korea and Japan, its prevalence in the general population was also high at 1.00%-1.72% in these countries ^(13)^. Although the discovery of this susceptibility gene was expected to clarify the pathogenetic mechanisms underlying MMD, gene-targeting experiments have not yet reproduced moyamoya vasculopathy ^(14), (15)^; therefore, the underlying mechanisms remain unclear despite this genomic breakthrough ^(16), (17)^. In addition, apart from its predominance in East Asians being associated with a high rate of *RNF213* mutations, the reasons for the other predominant conditions (females, children, young to middle-aged adults, and anterior circulation) remain unclear ^(5)^. Therefore, further studies are needed to elucidate the pathogenetic mechanisms of MMD.

As MMD and MMS have the same vascular lesions despite differences in their original pathogenesis ^(2)^, they may share a common trigger for the development of vascular lesions. We herein consider a common trigger from a novel perspective on blood flow dynamics. We focused on flow velocity and proposed increased flow velocity as a common trigger for the development of moyamoya vasculopathy. We discussed the rationale for this proposal based on supportive evidence from previous studies on blood flow dynamics.

## Search for a Common Trigger in Diseases Complicated by MMS

### 1. SCD

SCD has been identified as one of the most common causes of stroke in children worldwide ^(18)^. Moyamoya vasculopathy has been detected in approximately 40% of SCD patients with stroke history and is associated with a higher risk of recurrence with treatment resistance ^(19)^. Previous studies using transcranial Doppler (TCD) on SCD patients revealed that increased flow velocity (>200 cm/sec) in the middle cerebral arteries (MCAs) was predicted to be associated with the onset of stroke ^(20), (21)^. The amelioration of anemia with blood transfusions reduced the risk of stroke in SCD patients ^(22)^, and was accompanied by reductions in increased flow velocity ^(23)^ and cerebral blood flow ^(24)^. Magnetic resonance angiography examinations on SCD patients with increased flow velocity revealed that most patients did not have stenosis in the intracranial arteries ^(25)^. Therefore, increased flow velocity in SCD patients appears to be a reversible reaction depending on compensatory blood flow increases due to severe anemia. In contrast, transfusions for SCD patients with moyamoya vasculopathy do not sufficiently reduce the risk of stroke, suggesting that moyamoya vasculopathy is an irreversible change ^(19)^. These findings indicate that increased flow velocity occurs prior to rather than after the development of moyamoya vasculopathy, and, thus, may be a candidate trigger for vasculopathy. We herein focused on flow velocity and reviewed the literature to establish whether it is increased in other diseases complicated by MMS.

### 2. Down syndrome

MMS is one of the major causes of cerebral ischemia and stroke in Down syndrome ^(26)^. A previous study that assessed MCA flow velocity in Down syndrome demonstrated that peak systolic velocity was significantly higher (p = 0.04) in patients (139.75 ± 27.67 cm/sec) than in controls (123.89 ± 25.73 cm/sec) ^(27)^. The mechanisms underlying increased flow velocity in Down syndrome patients are yet to be determined; however, the following conditions have been suggested to increase flow velocity. The first condition is the high prevalence of anemia in Down syndrome (≥20%) ^(28)^. Anemia in Down syndrome may contribute to increased flow velocity, as described in the earlier section on SCD. The second condition is the high prevalence of sleep apnea in Down syndrome patients, which is estimated to be 50%-100% in childhood and nearly 100% in adulthood due to anomaly-related airway obstructions ^(29)^. Sleep apnea has been shown to increase flow velocity in the intracranial arteries ^(30)^, which is regarded as a reactive adaptation due to apnea-related hypercapnia ^(31)^. Flow velocity may increase in Down syndrome accompanied by anemia and/or sleep apnea.

### 3. Graves’ disease

Recent evidence indicates that increased thyroid function and elevated thyroid autoantibodies are associated with MMD, suggesting the importance of thyroid function in the development of moyamoya vasculopathy ^(32), (33)^. Although intracranial flow velocity is yet to be examined, previous studies reported increased flow velocity in the ophthalmic artery of Grave’s disease with severe ophthalmopathy ^(34), (35)^. Thyroid hormone-induced hyperdynamic circulatory reactions may increase flow velocity in systemic arteries, including the intracranial arteries.

### 4. Irradiation

MMS after radiation therapy is observed to be predominant in patients with optic glioma and in those treated with high-dose radiation. A previous study examining 58 patients with temporal radiation necrosis after irradiation showed a significantly higher flow velocity in patients (71.96 ± 12.08 cm/sec, p < 0.001) than in 29 matched controls (55.21 ± 6.02 cm/sec) in the chronic stage (4-5 years) ^(36)^. Although the mechanisms underlying increased flow velocity after irradiation have not yet been clarified, radiation-induced chronic inflammation may contribute to hyperemic reactions, leading to augmented flow and increased flow velocity.

### 5. Meningitis

While cerebral infarction due to inflammatory vasculopathy and hypercoagulability occurs in the acute or subacute phase of bacterial meningitis ^(37)^, MMS-induced stroke is occasionally associated with meningitis in the subacute or chronic phase ^(38)^. A TCD study on 94 patients with acute bacterial meningitis detected increased flow velocity (>150 cm/s) in 41 patients (43%) with a significantly higher risk of ischemic stroke and poorer outcomes (p < 0.001) than other patients ^(39)^. Arterial narrowing was confirmed via MR, CT, or conventional angiography in 9 out of 20 patients with increased flow velocity ^(39)^. Therefore, some meningitis patients exhibited increased flow velocity without stenotic changes, suggesting the presence of increased flow velocity prior to the development of moyamoya vasculopathy. Similar to irradiation, meningitis-induced chronic inflammation may contribute to increased flow velocity through a hyperemic reaction.

## Increased Flow Velocity in Predominant Conditions of MMD

### 1. Females

While a female predominance has been observed in both Asians and Caucasians, it appears to be more prominent in Caucasians (2.9- to 4.3-fold) ^(40), (41)^ than in East Asians (1.8- to 1.9-fold) ^(42), (43)^. A study on TCD flow velocity in healthy children showed that flow velocity in MCAs was found to be significantly higher (p = 0.005) in girls (89 ± 16 cm/s) than in boys (75 ± 16 cm/s) ^(44)^. Reference data from adult volunteers also demonstrated that flow velocity was significantly higher in females than in males aged between 20 and 59 years old ^(45)^. Therefore, females were determined to have a higher flow velocity than males of the same age from children to middle-aged adults.

### 2. Children

One of the most characteristic features of MMD is that it is a major cause of pediatric stroke ^(46), (47)^. Previous studies reported a peak age for the onset of MMD among 5-9 years old in East Asians ^(42), (48)^. Data on MCA flow velocity in 220 Japanese normal controls aged between 0 and 30 years old demonstrated that it increased with age from 0 years, peaked at 4-7 years (96.5 ± 15.6 cm/s at MCAs), and then decreased with age to 20 and 30 years (64.0 ± 11.5 cm/s at MCAs) ^(49)^. Accordingly, the peak age of flow velocity precedes and partially overlaps the peak age of the onset of MMD. This similar age dependence of flow velocity was reported in children in Europe. A TCD study on 112 healthy children younger than 18 years showed that maximal values were recorded at the age of 4-10 years ^(50)^. Consequently, flow velocity physiologically increased in children at the susceptible age for MMD.

### 3. Young to middle-aged adults

In addition to the sharp peak in childhood, a broad peak for the onset of MMD has been reported in adults in their 30s-40s. Flow velocity is not higher in these age groups than in other age groups under basic physiological conditions. An experimental study investigated the influence of age on intracranial blood flow regulation during sympathetic activation using the cold pressor test ^(51)^. Mean flow velocity in MCAs significantly increased (from 63 to 75 cm/s) in young adults in their 20s during immersion in ice-cold water, whereas no apparent increase (from 61 to 63 cm/s) was observed in older adults in their 60s ^(51)^. These findings indicated that young adults are susceptible to sympathetic activation and a subsequent increase in flow velocity. However, further studies are needed to further examine the effects and role of sympathetic activation on flow velocity in order to elucidate the precise mechanisms underlying predominancy in young to middle-aged adults.

### 4. The anterior circulation

The involvement of the anterior circulation is common in moyamoya vasculopathy, while that of the posterior circulation occurs in a delayed manner ^(52)^. In comparisons of flow velocity in intracranial arteries, previous studies demonstrated that flow velocity was higher in the anterior circulation than in the posterior circulation. TCD measurements in 106 normal volunteers revealed that flow velocities were 58 ± 15.6 and 39 ± 9.9 cm/s in MCAs (M1 segment) and the posterior cerebral arteries (PCAs: P1 segment), respectively ^(53)^. Another TCD study on 112 healthy children demonstrated that flow velocities were 81-97 and 50-57 cm/s in MCAs and PCAs, respectively ^(50)^. Accordingly, flow velocity is approximately 1.5-fold greater in the anterior circulation than in the posterior circulation under normal conditions.

## Detection of Increased Flow Velocity in MMD

As described earlier, flow velocity increases in several diseases complicated by MMS as well as under predominant conditions for MMD (Table 1), suggesting a relationship between flow velocity and susceptibility to moyamoya vasculopathy. If increased flow velocity is a common trigger for the development of moyamoya vasculopathy, it must be detected in MMD patients. However, difficulties have been associated with identifying increased flow velocity as a cause, not a result, of MMD because symptomatic patients have already developed steno-occlusive lesions, which secondarily alter flow velocity. Flow velocity in the intracranial major arteries is commonly measured via TCD. In TCD measurements, arterial stenosis alters flow velocity in the opposite direction according to the degree of stenosis. Flow velocity increases as the vascular cross section becomes smaller according to the laws of physics, but decreases in reverse when stenosis becomes very severe for near-occlusion ^(54), (55)^. TCD studies on MMD patients demonstrated that flow velocity increased in terminal internal carotid arteries (ICAs) and proximal MCAs with moderate to severe stenosis (Suzuki stages II-III) and then decreased in those with severe to near-occlusive stenosis (Suzuki stages III-IV) ^(56), (57), (58)^. Therefore, increased flow velocity in the majority of MMD cases has been attributed to the stenotic changes of moyamoya vasculopathy. However, an increase in flow velocity has been suggested in MMD premorbid conditions. A TCD study has reported that non-involved, namely, angiographically normal, intracranial arteries had significantly higher velocities in MMD patients (98.46 ± 20.38 cm/sec in MCAs, p < 0.05) than in age-matched controls (60.89 ± 13.61 cm/sec in MCAs) ^(59)^. A case-control study on vascular morphology and hemodynamics analyzed using computational fluid dynamics (CFD) revealed that simulated flow velocity and shear stress were both maximal in the internal carotid bifurcation and were higher in MMD patients than in controls, even though actual measured flow velocity was lower in MMD patients ^(60)^. This study also demonstrated that the ICAs of MMD patients were significantly shorter and less tortuous than those of normal controls ^(60)^. Consistent with this finding, a review showed that the affected ICA appeared to be less tortuous than the non-affected ICA in unilateral MMD patients using the parameter of the cavernous-supraclinoid angle of ICA ^(61)^. Therefore, shorter and lesser tortuous ICAs may contribute to smaller reductions in flow velocity, resulting in the maintenance of a higher flow velocity at the end of ICAs.

**Table 1.** Increased Flow Velocity in Moyamoya Syndrome, Predominant Conditions for Moyamoya Disease (MMD), and Non-Involved Arteries of MMD.

	Artery	Flow velocity	Cause	Reference
**Sickle cell disease**	MCA	>200 cm/sec is a risk factor for stroke	Anemia	20, 21
**Down syndrome**	MCA	Significantly higher than controls	Anemia, apnea?	27
**Graves’ disease**	Ophthalmic A	Significantly higher than controls	Hormonal	34, 35
**Irradiation**	MCA	Significantly higher than controls	Inflammation?	36
**Meningitis**	MCA	>150 cm/sec is a risk factor for stroke	Inflammation?	39
**Females**	MCA	Significantly higher than males	Original	44, 45
**Children**	MCA	Higher than other ages*	Original	49, 50
**Young/middle-aged adults**	MCA	Higher than older adults under a sympathetic stimulation	Original	51
**Anterior circulation**	MCA	Higher than PCA*	Original	50, 53
**MMD**	Non-involved A	Significantly higher than controls	Idiopathic	59

A: artery, MCA: middle cerebral artery, PCA: posterior cerebral artery. *not statistically examined in the cited literature

## Vascular Biological Considerations for the Trigger Effect of Increased Flow Velocity in the Development of Moyamoya Vasculopathy

**Figure 1.**
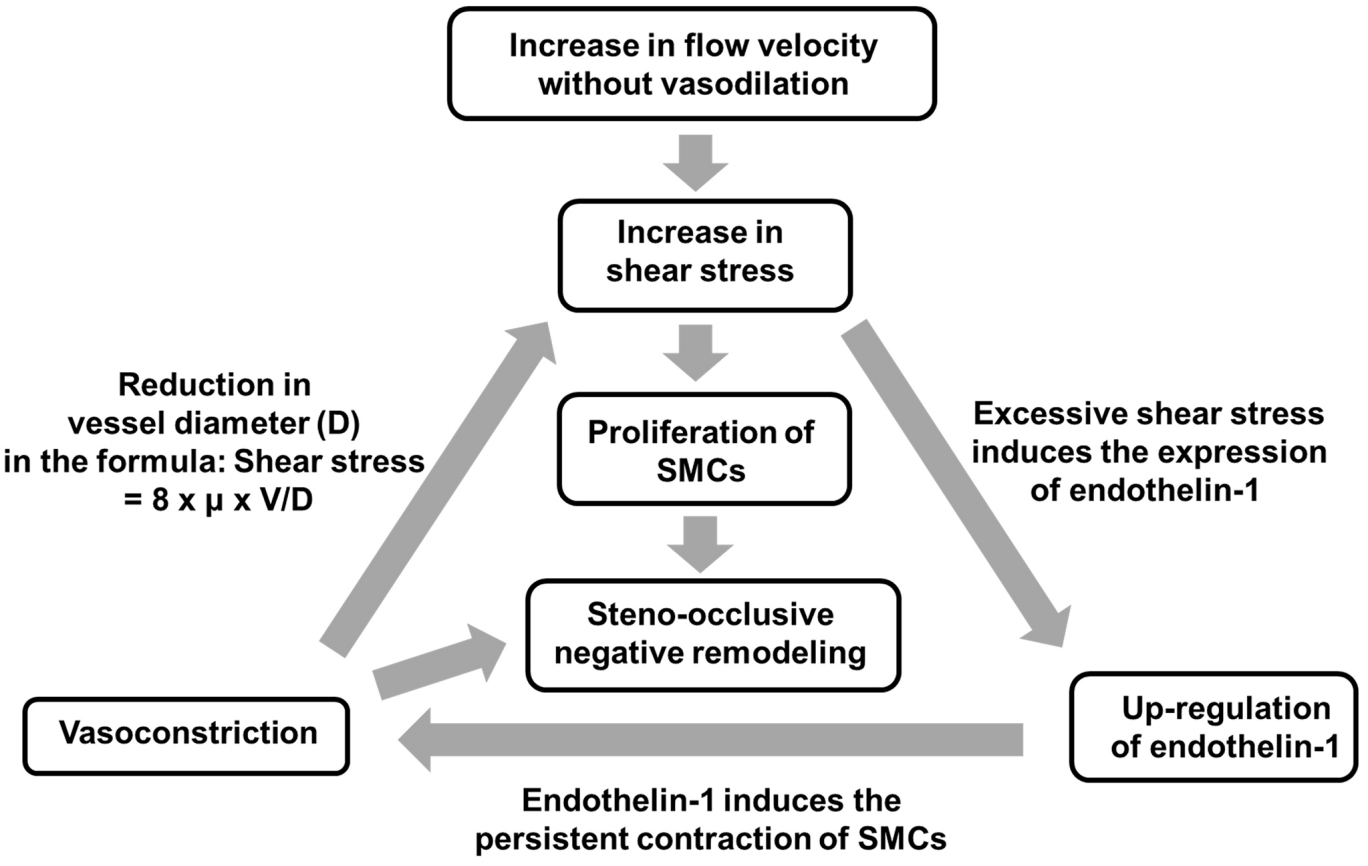
Vascular biological considerations for increased flow velocity in the development of steno-occlusive negative remodeling. An increase in flow velocity may activate a vicious circle of elevated shear stress, the upregulation of endothelin-1, and vasoconstriction with the proliferation of smooth muscle cells.

Flow velocity affects shear stress, a biomechanical vasoactive factor that is expressed with the following equation: shear stress = 8 × μ × V/D, where μ, V, and D indicate blood viscosity, flow velocity, and vascular diameter, respectively ^(62)^. Therefore, if flow velocity increases without sufficient vasodilation, it then strengthens shear stress on the endothelium. An arteriovenous fistula (AVF) animal model has been used to investigate vascular remodeling under the condition of increased flow and increased shear stress. Chronic high shear stress with intermittent short-duration exposure to low wall shear stress can cause intimal thickening with the migration and proliferation of smooth muscle cells in the AVF model ^(63)^. A study with cultured arterial endothelial cells revealed that increases in shear stress up to 90-120 dyn/cm2 attenuated the endothelial release of NO and upregulated the expression of endothelin-1, suggesting that excessively increased shear stress induced endothelin-1-induced vasoconstriction ^(64)^. Therefore, if increased flow velocity without vasodilation strengthens shear stress, shear stress may, in turn, increase itself more in a vicious circle of elevated shear stress, the upregulation of endothelin-1, and vasoconstriction (Figure 1). In other words, increased flow velocity may have a trigger effect on the development of moyamoya vasculopathy. Radiological and pathological analyses of MMD patients revealed that steno-occlusive vascular lesions were accompanied by a decrease in the outer diameter ^(65), (66), (67)^, called “negative remodeling.” Although a number of issues have yet to be clarified from the viewpoint of vascular biology, steno-occlusive negative remodeling may be relevant to the vicious cycle of elevated shear stress, the upregulation of endothelin-1, and vasoconstriction with the proliferation of smooth muscle cells (Figure 1). Rapid progression of vascular stenosis sometimes observed in MMD and/or MMS could be explained by acceleration of the vicious cycle reactions.

## Pathogenetic Role of *RNF213* in the Development of Moyamoya Steno-occlusive Vasculopathy

Although a relationship has been noted between the *RNF213* mutation and moyamoya steno-occlusive vasculopathy ^(68), (69)^, the pathogenetic role of *RNF213* in the development of vascular lesions remains unclear. Gene-targeting experiments with *RNF213*-deficient mice demonstrated that a deficiency in *RNF213* was not sufficient to reproduce steno-occlusive vasculopathy ^(14), (15)^. These findings imply that the *RNF213* mutation initiates an indirect pathway to complete vascular remodeling that is not easily reproduced in rodents. A clinical study to investigate the relationship between a variant of *RNF213* and the morphology of ICAs demonstrated that the tortuosity of ICAs was significantly lower in the *RNF213*-mutant group than in the wild-type group ^(70)^. Since the CFD study cited in the previous section demonstrated that lower ICA tortuosity in MMD patients resulted in increased flow velocity/shear stress, particularly at terminal ICAs ^(60)^, less tortuosity in patients with the *RNF213* mutation may contribute to an increase in flow velocity/shear stress and may secondarily induce vascular remodeling. Although a relationship has been demonstrated between the *RNF213* mutation and vascular morphological changes, its reproducibility needs to be further examined using larger patient cohorts, and the possibility of other structural or functional phenotypes due to the *RNF213* mutation also needs to be considered.

## Comprehensive View on Pathogenetic Mechanisms of Moyamoya Vasculopathy

**Figure 2.**
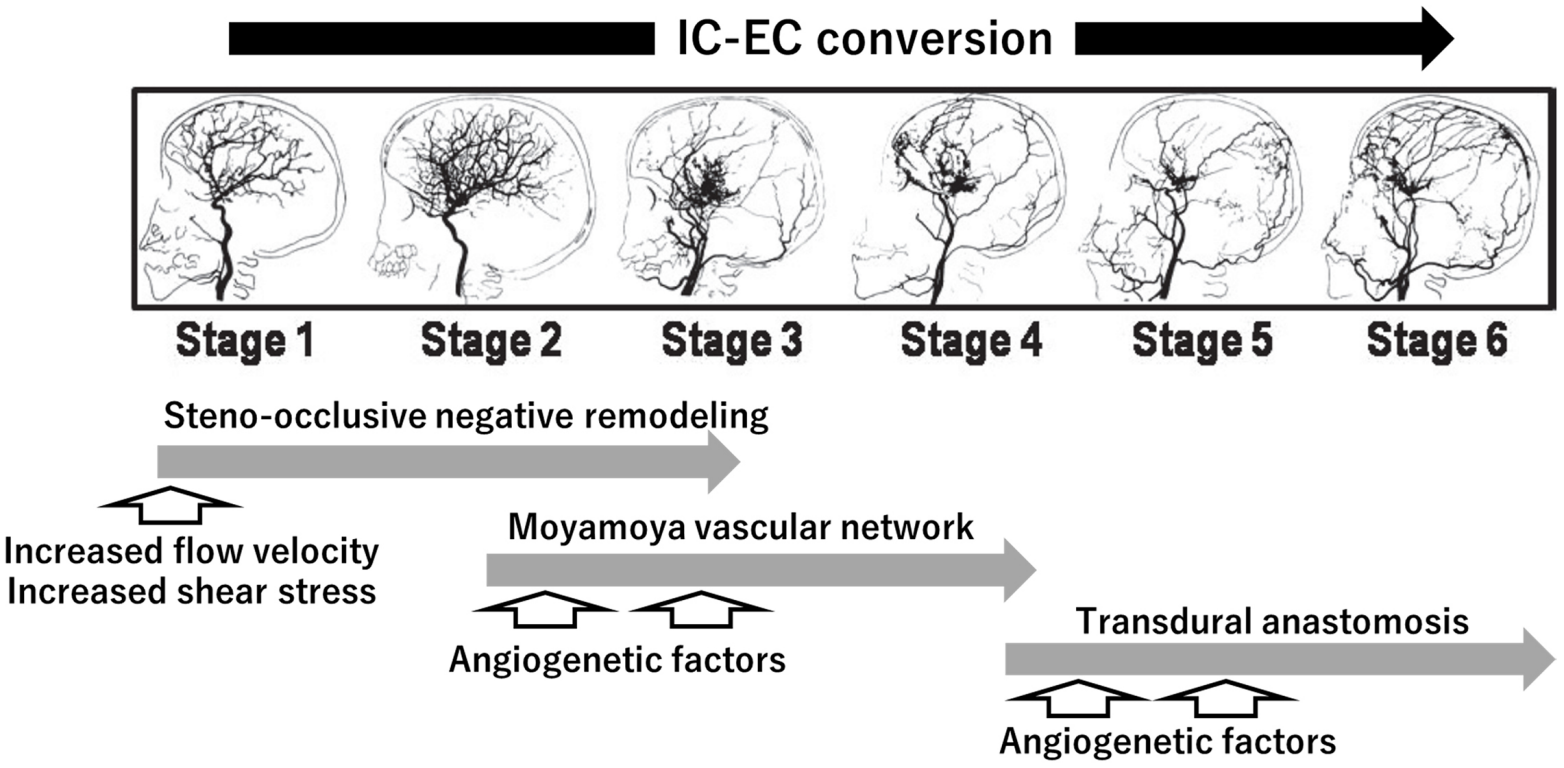
A comprehensive view of pathogenetic mechanisms underlying vascular lesions of MMD.

The vascular lesions of MMD are characterized by chronic progressive steno-occlusive lesions at the terminal portion of the ICA and the formation of a “moyamoya” vascular network at the base of the brain. ICAs and moyamoya vessels disappear with the progression of vascular lesions, and transdural anastomosis newly appears as a collateral vascular supply for the brain. Therefore, chronic progressive changes may be regarded as the gradual conversion of the cerebral vascular supply from the intracranial/IC system to the extracranial/external carotid (EC) system, or the so called “IC-EC conversion” (Figure 2) ^(71), (72)^. In the pathogenetic overview of chronic progressive steno-occlusive lesions showing the IC-EC conversion, we propose a novel perspective including the trigger effect of increased flow velocity. If flow velocity is increased for an idiopathic reason (MMD) or secondary reason (MMS) accompanied by the following conditions that are associated with an increased flow velocity, namely, child, female, sympathetic activation, anemia, apnea, and hormonal factors, it may strengthen shear stress under the conditions of insufficient vasodilation. This may, in turn, initiate the amplified process of lesion formation, leading to steno-occlusive negative remodeling (Figure 1). Thereafter, the gradual progression of cerebral ischemic changes may induce intrinsic compensatory responses with the aid of angiogenetic factors, represented by the moyamoya vascular network in the middle stages of MMD and transdural anastomosis in the late stages (Figure 2). The perspective including the trigger effect of increased flow velocity may be a rational explanation for the predominant conditions and initial process of the vascular remodeling of MMD.

## Limitations

This review has several limitations. First, there has been no direct evidence that increased flow velocity is a cause of progressive steno-occlusive lesions. Prospective studies of the very early stage of MMD and/or unilateral MMD could be useful to clarify the causal relationship between flow velocity and progression of moyamoya vasculopathy. Second, hemodynamic data, which we cited as indirect supportive evidence, were from individual studies on MMS or MMD with relatively small number of cases. Similar hemodynamic data are needed from large number of cases. Third, whether the characteristic arterial stenosis with outer diameter narrowing, or the so called “negative remodeling” in MMD, is the primary change or the secondary phenomenon in response to the altered flow velocity remains unclear. In light of the recent evidence that *RNF213* variant is closely associated with negative remodeling (outer diameter narrowing with vessel wall thinning) and enhanced flow velocity alteration by high-resolution magnetic resonance imaging/angiography, *RNF213* variant may render vessels more vulnerable to hemodynamic stress ^(73)^. Fourth, although this review focused on increased flow velocity for the pathogenetic mechanisms, others mentioned that the hemodynamic attenuation changes can likely trigger the development of MMD. A case report demonstrated that decrease in flow velocity in ICA was prior to de novo development of MMD after stereotactic radiosurgery for AVM in a patient with *RNF213* mutation ^(74)^. A study with *RNF213* knockout mice revealed that induced stenosis of bilateral common carotid arteries promoted further cerebral hypoperfusion ^(75)^. Therefore, future studies on hemodynamic pathogenesis for moyamoya vasculopathy should focus on not only increased flow velocity but also on decrease flow velocity.

## Conclusions and Future Perspectives

In this review, we have discussed increased flow velocity in several diseases complicated by MMS (SCD, Down syndrome, Graves’ disease, irradiation, and meningitis), the predominant conditions of MMD (females, children, young to middle-aged adults, and anterior circulation), and non-involved arteries in MMD (Table 1). Increased flow velocity may be a common trigger for the development of the vascular lesions of MMD. We anticipate reports of causative increased flow velocity in MMD patients worldwide, which will support its pathogenetic importance. Furthermore, we expect an animal model of MMD to be developed with measures to increase flow velocity, leading to the elucidation of the underlying pathogenetic mechanisms and the development of treatment options for MMD.

## Article Information

### Conflicts of Interest

None

### Author Contributions

Both authors were involved in the review design and collection of references. T.A. wrote the manuscript, M.F. checked the contents, and both approved the final manuscript.

### Disclaimer

Miki Fujimura is one of the Editors of JMA Journal and on the journal’s Editorial Staff. He was not involved in the editorial evaluation or decision to accept this article for publication at all.
